# Estrogenic Exposure Alters the Spermatogonial Stem Cells in the Developing Testis, Permanently Reducing Crossover Levels in the Adult

**DOI:** 10.1371/journal.pgen.1004949

**Published:** 2015-01-23

**Authors:** Lisa A. Vrooman, Jon M. Oatley, Jodi E. Griswold, Terry J. Hassold, Patricia A. Hunt

**Affiliations:** School of Molecular Biosciences, Center for Reproductive Biology, Washington State University, Pullman, Washington, United States of America; Stowers Institute for Medical Research, UNITED STATES

## Abstract

Bisphenol A (BPA) and other endocrine disrupting chemicals have been reported to induce negative effects on a wide range of physiological processes, including reproduction. In the female, BPA exposure increases meiotic errors, resulting in the production of chromosomally abnormal eggs. Although numerous studies have reported that estrogenic exposures negatively impact spermatogenesis, a direct link between exposures and meiotic errors in males has not been evaluated. To test the effect of estrogenic chemicals on meiotic chromosome dynamics, we exposed male mice to either BPA or to the strong synthetic estrogen, ethinyl estradiol during neonatal development when the first cells initiate meiosis. Although chromosome pairing and synapsis were unperturbed, exposed outbred CD-1 and inbred C3H/HeJ males had significantly reduced levels of crossovers, or meiotic recombination (as defined by the number of MLH1 foci in pachytene cells) by comparison with placebo. Unexpectedly, the effect was not limited to cells exposed at the time of meiotic entry but was evident in all subsequent waves of meiosis. To determine if the meiotic effects induced by estrogen result from changes to the soma or germline of the testis, we transplanted spermatogonial stem cells from exposed males into the testes of unexposed males. Reduced recombination was evident in meiocytes derived from colonies of transplanted cells. Taken together, our results suggest that brief exogenous estrogenic exposure causes subtle changes to the stem cell pool that result in permanent alterations in spermatogenesis (i.e., reduced recombination in descendent meiocytes) in the adult male.

## Author Summary

During the past several decades, the incidence of human male reproductive abnormalities such as hypospadias, undescended testicles, testicular cancer, and low sperm counts has increased. Environmental factors—and in particular, exposure to environmental estrogens—have been implicated as contributing factors and, indeed, developmental exposure to a range of estrogenic chemicals induces similar defects in male rodents. Given the wide variety of ‘weak’ estrogenic chemicals found in everyday products, understanding how estrogenic exposures affect sperm production has direct human relevance. Here we show that brief exposure of newborn male mice to exogenous estrogen affects the developing spermatogonial stem cells of the testis and this, in turn, permanently alters spermatogenesis in the adult. Specifically, estrogens adversely affect meiotic recombination, a process that is essential for the production of haploid gametes. Subtle changes in the levels of recombination increase the incidence of meiotic errors, resulting in the elimination of cells before they become sperm. Thus, in addition to their other potential effects on the developing brain and reproductive tract, our results suggest that estrogenic exposures can act to reduce sperm production by affecting the spermatogonial stem cell pool of the developing testis.

## Introduction

Over the past few decades, there has been increasing concern that sperm counts and quality are declining [1, 2]. In Denmark, for example, sperm counts have declined over time, and more than 40% of young Danish men have sperm counts in the range associated with infertility or decreased fertility [3–6]. These findings are echoed by reports from Japan, the United States, and other European countries [7–11]. In addition to changes in sperm counts, there has been a corresponding increase in the incidence of morphological abnormalities of male reproductive organs, including hypospadias and undescended testicles as well as an increased incidence of testicular cancer [12]. This constellation of male disorders is termed testicular dysgenesis syndrome (TDS) and is postulated to be developmental in origin.

Specifically, TDS was originally proposed to result from exposure of the developing male to maternally-derived or environmental estrogens [13]. Both correlative data from studies in humans and experimental studies using animal models have provided support for the hypothesis. Diethylstilbestrol (DES) is a potent synthetic estrogen that was prescribed to pregnant women from 1940 to the early 1970s. Men that were exposed in utero to DES have an increased incidence of cryptorchidism, underdeveloped testes, testicular cancer, low sperm counts, and decreased sperm quality [14–18]. Thus, the human DES experience demonstrates that fetal exposure to exogenous estrogens can induce TDS symptoms. More recent experimental studies have shown that exposure of the developing testis to potent estrogens or endocrine disrupting chemicals (EDCs) with estrogenic activity during either prenatal or early postnatal development can cause a reduction in testis weight and sperm counts in adult male rodents [19–23]. However, because testicular changes have not been a feature of all studies, the effect of exposures—at least for some chemicals—on the developing testis has remained controversial.

Estrogenic exposures can impact the developing brain, changing behavior and neuroendocrine function [24], as well as the reproductive tract, interfering with gamete maturation and delivery [25, 26]. The complexity of interacting and interdependent systems has made it difficult to pinpoint the mechanism(s) by which exogenous estrogens exert effects on the developing testis. Spermatogenesis is a complex process that is dependent upon both endocrine and paracrine signaling to accomplish the multiple cell divisions necessary to renew the spermatogonial stem cell (SSC) population and give rise to populations of differentiating cells that ultimately produce sperm. Exogenous estrogens have been reported to affect both the steroid hormone-producing Leydig cells and the Sertoli cells that are essential for spermatogenesis [27–31], but direct effects on germ cells remain poorly understood.

Meiosis is the specialized cell division that is essential for the reduction of diploid germ cells to haploid gametes during gametogenesis. In female mice, BPA exposure beginning prior to the onset of meiosis and continuing through meiotic prophase (11–18.5 dpc) significantly alters the events of meiotic prophase, increasing recombination or crossover levels (scored as the number of foci of the crossover-associated protein, MLH1) and resulting in the production of aneuploid eggs [32, 33]. Although the available evidence from studies in rodents suggests that developmental exposures to exogenous estrogens can impact spermatogenesis in adulthood [20–23], the potential meiotic effects of these exposures have not been evaluated. To determine if an exposure comparable to that in the female (i.e., corresponding to the time of meiotic commitment and onset) affects male meiosis, we exposed male mice to BPA or to the strong synthetic estrogen, ethinyl estradiol (EE) during neonatal development. As in the female, we found that exposure influenced meiotic prophase in the male. In contrast to the female, however, where BPA exposure increased meiotic recombination levels, significantly reduced levels were evident in the first wave of meiotic cells from males exposed to BPA or EE by comparison with placebo males. Remarkably, the effect persisted into adulthood long after the exposure ended, with significantly reduced recombination levels in BPA and EE males by comparison with placebo males. Furthermore, our investigation identified ‘estrogen-sensitive’ mouse strains, as well as one inbred strain that was resistant to the meiotic effects of exogenous estrogens. Lastly, the transplantation of SSCs from exposed males to the testes of unexposed recipients demonstrated that the recombination phenotype results from permanent alterations to the SSCs. Taken together, our data provide evidence that exposure to exogenous estrogens during testis development can induce permanent alterations to the SSC population that act to reduce spermatocyte survival in the adult.

## Results

### Neonatal exposure to estrogenic chemicals permanently reduces meiotic recombination levels

To test the hypothesis that neonatal estrogenic exposure disrupts meiosis, newborn male mice were given single daily oral doses of BPA or vehicle-only placebo from 1–12 days postpartum (dpp). In addition, because spermatogenic impairment has been postulated to result not simply from BPA but from estrogenic exposures in general (e.g., [19]), we also tested the effect of exposure to the synthetic estrogen used in birth control pills and hormone replacement therapy, ethinyl estradiol (EE). EE is not only a potent estrogen frequently used as a positive control in studies of endocrine disruptors, but is also a common water contaminant that remains detectable even after sewage treatment [34, 35]. Because strain differences in estrogen sensitivity have been reported [36], we evaluated both outbred CD-1 and inbred C57BL/6J (B6) males. To assess effects on synapsis and recombination, meiotic analyses were performed on 20 dpp males by immunostaining surface spread preparations of meiotic cells with antibodies for SYCP3 and MLH1. SYCP3 is a component of the synaptonemal complex (SC), and MLH1 is a DNA mismatch repair protein that localizes to the large majority of sites of meiotic exchange [37]. Perturbations in synapsis were not observed in either strain (S1 Table), but MLH1 levels were significantly reduced in BPA and EE exposed CD-1 males (Table 1, S1 Fig.). Mean MLH1 counts for juvenile CD-1 males were 22.27 ± 0.12, 21.50 ± 0.10, 21.87 ± 0.10, and 20.85 ± 0.10 for placebo, 20 ng BPA, 500 ng BPA, and 0.25 ng EE-exposed, respectively (p<0.0001). In contrast, no differences were detected in B6 males with any exposure.

**Table 1:** Recombination rates* in placebo and exposed males.

	**Exposure (ng/g/day)**	**CD-1**	**C57BL/6J**	**C3H/HeJ**	**C3H/HeJ x C57BL/6J F1**
20 dpp	Placebo	22.27 ± 0.12^a^	22.95 ± 0.16	21.75 ± 0.14^a^	23.69 ± 0.23^a^
	20 ng BPA	21.50 ± 0.10^b^	22.97 ± 0.15	—	—
	500 ng BPA	21.87 ± 0.10^c^	23.02 ± 0.14	—	—
	0.25 ng EE	20.85 ± 0.10^d^	23.01 ± 0.15	20.55 ± 0.11^b^	23.14 ± 0.17^b^
12 weeks	Placebo	24.26 ± 0.18^a^	24.02 ± 0.16	22.98 ± 0.13^a^	24.80 ± 0.14^a^
	20 ng BPA	23.22 ± 0.16^b^	24.39 ± 0.19	—	—
	500 ng BPA	22.95 ± 0.15^b^	23.89 ± 0.19	—	—
	0.25 ng EE	21.72 ± 0.13^c^	23.80 ± 0.16	21.98 ± 0.10^b^	24.44 ± 0.15^b^
1 year	Placebo	24.49 ± 0.18^a^	—	—	—
	20 ng BPA	23.14 ± 0.20^b^	—	—	—
	500 ng BPA	23.40 ± 0.22^b^	—	—	—
	0.25 ng EE	23.13 ± 0.19^b^	—	—	—

*Mean MLH1 ± SEM for 25–30 pachytene cells per male, with 5–12 males/group.

^a-d^ Exposure within a strain and age were compared by one-way ANOVA (CD-1, C57BL/6J) or one-tail t-test (C3H/HeJ, C3H/B6 F1). Superscript letters denote significant differences as determined by a Newman-Keuls post hoc test (p<0.05); like letters indicate no difference.

— Denotes experimental group was not performed.

To determine if the reduction in meiotic recombination induced by neonatal exposure was transient, littermates of juvenile males used in initial experiments were aged and analyzed as adults (Table 1; S1 Fig.). MLH1 levels in BPA or EE exposed CD-1 males assessed at 12 weeks were significantly reduced by comparison with placebos. Mean MLH1 counts were 24.26 ± 0.18, 23.22 ± 0.16, 22.95 ± 0.15, and 21.72 ± 0.13 for placebo, 20 ng BPA, 500 ng BPA, and 0.25 ng EE-exposed, respectively (p<0.0001). Further, the effect persisted throughout the reproductive lifespan, as evidenced by the fact that a similar reduction was observed in CD-1 males aged to one year, with mean MLH1 counts of 24.49 ± 0.18, 23.14 ± 0.20, 23.40 ± 0.22, and 23.13 ± 0.19 for placebo, 20 ng BPA, 500 ng BPA, and 0.25 ng EE-exposed, respectively (p<0.0001). As in the analysis of 20 dpp males, no difference in MLH1 levels was observed with any exposure on the B6 strain.

### Sensitivity is influenced by genetic background

To determine if the effect of neonatal estrogenic exposure on meiotic recombination was limited to outbred strains, we tested the effect of neonatal EE exposure on the inbred C3H/HeJ (C3H) strain (Table 1; S1 Fig.). Like CD-1 mice, exposed male C3H mice had significantly reduced MLH1 levels, with mean counts of 21.75 ± 0.14 for placebo and 20.55 ± 0.11 for EE-exposed at 20 dpp (p<0.0001), and 22.98 ± 0.13 for placebo and 21.98 ± 0.10 for EE-exposed at 12 weeks old (p<0.0001).

The lack of meiotic abnormalities in exposed B6 males was surprising since B6 males were previously reported to be ‘estrogen sensitive’ [36]. To investigate this further, we mated C3H females (‘sensitive strain’) with B6 males (‘resistant strain’) and evaluated F1 hybrid males (Table 1; S1 Fig.). MLH1 counts were 23.69 ± 0.23 for placebo and 23.14 ± 0.17 for EE-exposed males at 20 dpp (p<0.05), and 24.80 ± 0.14 for placebo and 24.44 ± 0.15 for EE-exposed males at 12 weeks old (p<0.05). At both ages, F1 males exhibited a reduction in exchanges in response to EE exposure, demonstrating that ‘resistance’ is not a simple dominant effect.

Estrogen sensitivity has been reported to be influenced by uterine environment [38]. Thus, it remained possible that the estrogen sensitivity in C3H/B6 F1 hybrid males was due to environmental (e.g., development in a ‘sensitive’ C3H uterine environment) rather than genetic factors. To determine if the estrogen insensitivity of B6 males could be ‘reprogrammed’ by the uterine environment we transferred one-cell B6 embryos to a ‘sensitive’ CD-1 uterine environment and tested the effect of exposure on the resultant males. Although embryo transfer to the CD-1 uterine environment slightly affected baseline MLH1 levels in B6 males, neonatal exposure to estrogens did not elicit an effect (S2 Table). Thus, the data from embryo transfer experiments support the hypothesis that the insensitivity of B6 males to estrogen is a reflection of genetic differences.

### Estrogenic exposure increases the frequency of recombination failure

**Figure 1:**
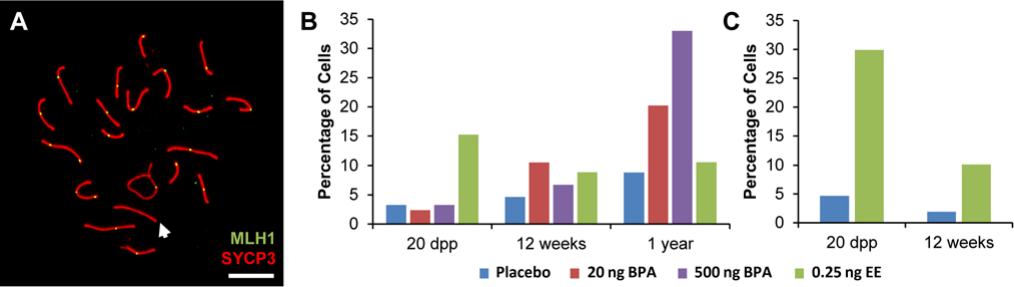
Analysis of pachytene cells from CD-1 and C3H males. A) Representative spermatocyte with an SC lacking an MLH1 focus (white arrow) from an adult C3H male orally exposed to 0.25 ng EE from 1–12 dpp. MLH1 = green, SYCP3 = red. B, C) The frequency of cells with an SC lacking MLH1 in placebo and exposed males analyzed at 20 dpp, 12 week-old, and 1 year-old CD-1 males (B) and 20 dpp and 12 week-old C3H males (C). Differences were tested by Chi-square analysis. For CD-1, X^2^ = 51.70 (p<0.0001) at 20 dpp, X^2^ = 3.57 (p>0.05) at 12 weeks, and X^2^ = 1.56 (p>0.05) at 1 year-old. For C3H, X^2^ = 32.87 (p<0.0001) at 20 dpp, and X^2^ = 13.95 (p<0.001) at 12 weeks.

To better understand the effect of estrogenic exposures on recombination, we characterized the distribution of the sites of exchange in pachytene cells from exposed males. We conducted two types of analyses. First, we determined the basis for the difference in the number of MLH1 foci per cell between exposed and control males. For all time points, the reduction in MLH1 foci in exposed CD-1 and C3H males could be attributed to a decreased frequency of SCs with two MLH1 foci and a corresponding increase in SCs with a single focus (S3 Table). Second, we asked whether estrogenic exposure increased the likelihood that cells would contain at least one SC lacking an MLH1 focus; i.e., a “crossover-less” SC (Fig. 1A). Indeed, we found that, on both genetic backgrounds, neonatal EE exposure increased the frequency of such cells in juvenile males. Specifically, cells containing an SC without an MLH1 focus increased in frequency from 2–3% in placebo to 15% in EE-exposed males for CD-1 (Fig. 1B; p<0.0001), and from approximately 5% in placebo to 30% in EE-exposed males for C3H (Fig. 1C; p<0.0001). The results for adult males varied between the two genetic backgrounds—no significant differences were observed in adult CD-1 males (Fig. 1B), but in adult C3H males, cells containing an SC without an MLH1 focus increased in frequency from approximately 2% for placebo to 10% in EE-exposed males (Fig. 1C; p<0.0005).

**Figure 2:**
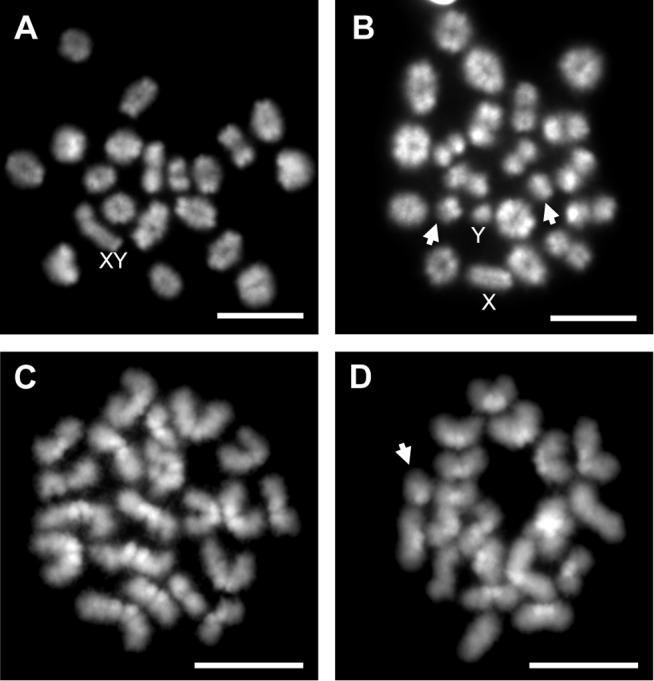
Analysis of metaphase I (MI) and metaphase II (MII) cells in C3H spermatocytes from placebo and exposed males. A) Normal MI spermatocyte from a placebo male. B) MI spermatocyte with small autosomal univalents (arrows) and sex chromosome univalents (X and Y) from an EE-exposed male. C) Normal MII spermatocyte from a placebo male. D) MII spermatocyte with an extra chromatid (arrow) from an EE-exposed male. Scale bars represent 10 μm.

Because recombination failure results in unpartnered univalents at metaphase I that trigger cell death [39], we analyzed the frequency of univalents at metaphase I (MI) in the strain that exhibited a significant increase in crossover-less SCs, C3H. As predicted, neonatal EE exposure increased the frequency of abnormal MI cells in adult males (Table 2, p<0.005; Fig. 2A and B). In placebo males, 3.90% of cells had a single pair of autosomal univalents, and this increased to 10.34% in EE-exposed males. In placebos, all autosomal univalents involved small chromosomes. With the exception of a single mid-sized pair of autosomal univalents, this was also true in EE-exposed males. In addition, the frequency of sex chromosome univalents was high in placebo males (6.49% of cells), but significantly increased in EE-exposed males (23.28% of cells). To determine if cells containing univalents were effectively eliminated, we conducted a similar analysis of cells at metaphase II (MII). No abnormalities were observed in MII cells from placebo males, but 5.13% of MII cells were abnormal in EE-exposed males; 3 cells contained prematurely separated sister chromatids, 2 had an extra chromatid, and the remaining cell was missing a chromatid. (Table 3, Fig. 2C and D).

**Table 2:** Analysis of metaphase I in placebo and exposed C3H males.

	Total Normal*	**Total Abnormal**	**Univalent Autosome**	**Univalent XY**
Placebo	69 (89.61)	8 (10.39)	3 (3.90)	5 (6.49)
0.25 ng EE	82 (70.69)	34 (29.31)	12 (10.34)	27 (23.28)

*Data represent number of cells and percentage in parentheses from 20–27 MI cells per animal from 3 placebo and 5 EE-exposed C3H males; X^2^ = 8.65, p<0.005 for comparison of normal and abnormal cells.

**Table 3:** Analysis of metaphase II in placebo and exposed males.

	Total Normal*	**Total Abnormal**	**Extra chromatid**	**Missing chromatid**	**Premature separation**
Placebo	64 (100.00)	0 (0.00)	0 (0.00)	0 (0.00)	0 (0.00)
0.25 ng EE	111 (94.87)	6 (5.13)	2 (1.71)	1 (0.85)	3 (2.56)

*Data represent number of cells and percentage in parentheses from 20–25 MII cells per animal from 3 placebo and 5 EE-exposed C3H males.

### Neonatal estrogenic exposure alters spermatogonial stem cells

**Figure 3:**
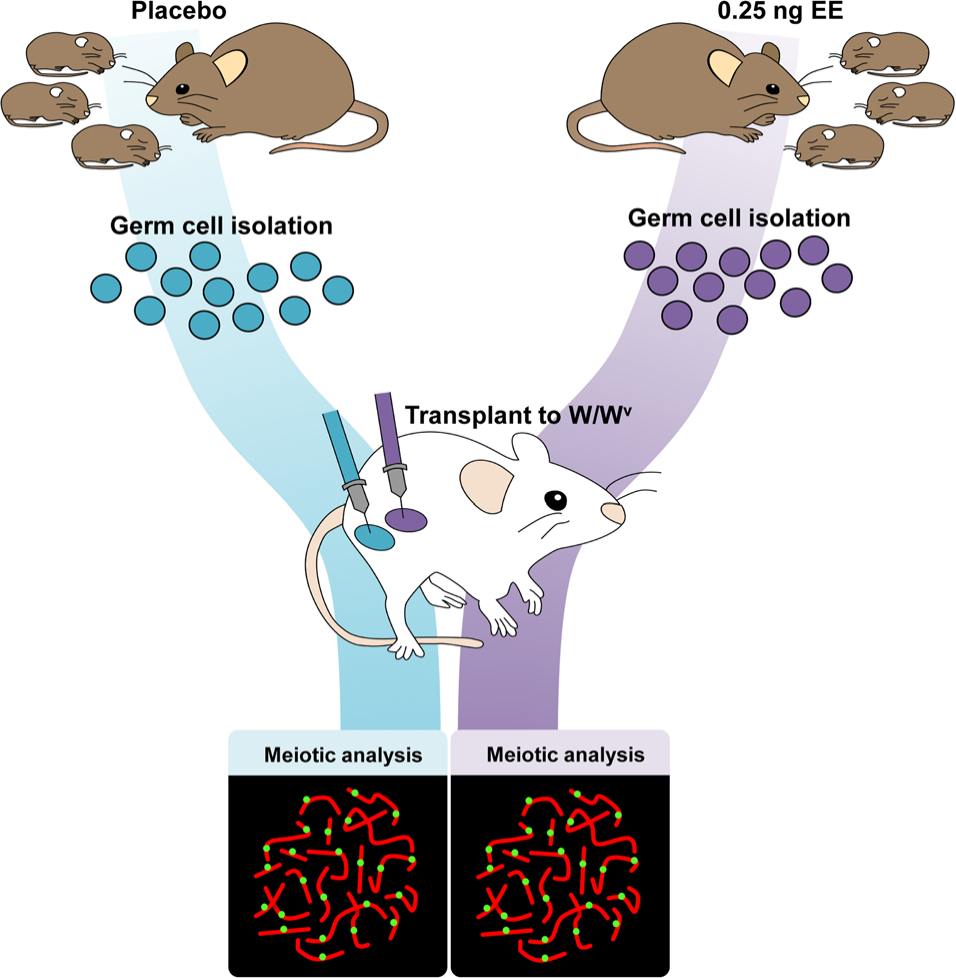
Germ cell transplantation experimental design. C3H male pups from timed-pregnant C3H females were orally exposed to placebo or 0.25 ng/g/day EE from 1–12 dpp. At 13 dpp, testes were pooled per exposure group, germ cells were isolated, and transplanted into W/W^v^ adult males. Placebo cells were injected into one testis, and EE-exposed cells into the contralateral testis. Different letters and numbers represent different transplantation days and individuals, respectively. Meiotic analyses occurred 8 weeks post-transplantation.

Because spermatogenesis is continuous, the permanent reduction in recombination observed in exposed males suggests that estrogens affect one or more cell populations of the developing testis. To determine if neonatal EE exposure alters the somatic or germ cell lineage, we performed germ cell transplantation experiments. C3H males were exposed to placebo or EE from 1–12 dpp. On 13 dpp, germ cells were isolated and transplanted into the seminiferous tubules of recipient W/W^v^ males (Fig. 3). W/W^v^ males lack endogenous germ cells, but maintain a somatic environment capable of supporting spermatogenesis, allowing colonization of transplanted donor cells and initiation of spermatogenesis from them [40]. MLH1 levels were significantly reduced in meiocytes resulting from the transplantation of SSCs from EE-exposed males by comparison to placebo (Table 4, p<0.0001). Mean MLH1 counts were 24.11 ± 0.25 and 22.58 ± 0.22 for transplants from placebo or EE-exposed males, respectively. On an individual basis, five of six W/W^v^ recipient males exhibited a reduction in MLH1 levels in the testis transplanted with cells from EE exposed males by comparison with the contralateral testis transplanted with cells from placebo exposed males (p<0.05 for all males). Interestingly, MLH1 counts in cells from testes transplanted with placebo cells were higher than those in intact placebo exposed adult C3H males (Table 1, adult C3H MLH1 = 22.98 ± 0.13), indicating a slight effect of the transplant procedure on recombination.

**Table 4:** Recombination in transplanted males.

**W/W^v^ male^1^**	**Placebo transplant^2^**	**0.25 ng EE transplant^3^**
A1	23.91 ± 1.01	22.23 ± 0.47*
A2	23.83 ± 0.33	22.73 ± 0.35*
B1	23.10 ± 0.52	23.72 ± 0.50
B2	23.20 ± 0.47	21.67 ± 0.39*
C1	25.89 ± 0.41	23.85 ± 0.41*
C2	24.83 ± 0.43	23.00 ± 0.61*
Pooled	24.11 ± 0.21	22.84 ± 0.19*
	n = 157	n = 167

^1^Recipients with different letters were transplanted on different days.

^2^Germ cells from placebo-exposed donors in one testis.

^3^Germ cells from EE-exposed males in the contralateral testis.

Values represent mean MLH1 ± SEM.

n = number of cells analyzed.

*indicates significant difference between exposed and placebo cells in one-tailed t-test (p<0.05)

### Exposure reduces meiotic recombination without affecting synapsis or double strand break formation

**Figure 4:**
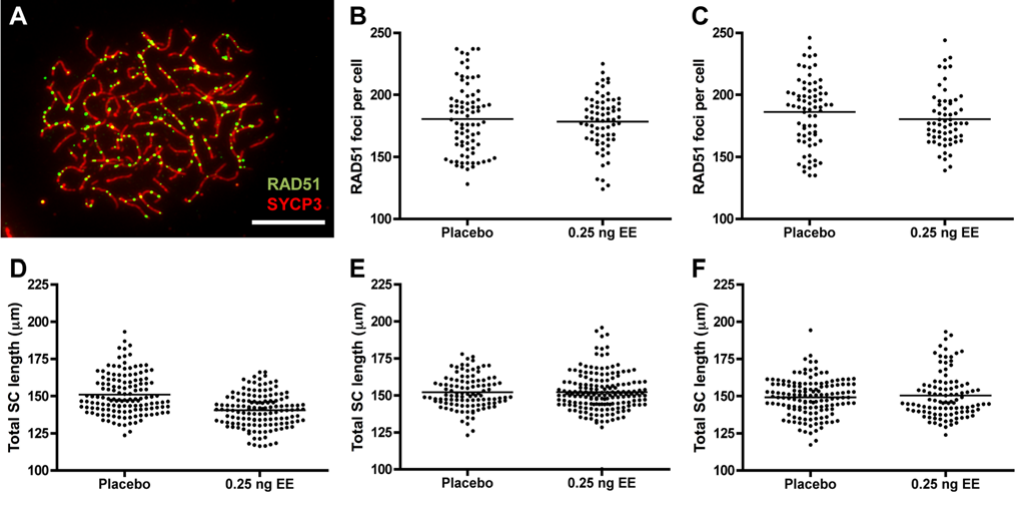
RAD51 foci counts and SC length measurements in placebo and exposed CD-1 males. A) RAD51 foci in zygotene cell from 20 dpp CD-1 male exposed to 0.25 ng/g/day EE. RAD51 = green, SYCP3 = red, scale bar = 10 μm. B, C) Mean RAD51 counts in placebo and EE-exposed CD-1 males at 20 dpp (B) and 12 weeks of age (C). Data points represent RAD51 foci values from 20–25 zygotene cells from 3–4 males from Table 1. D-F) Total autosomal SC length (μm) in pachytene cells from placebo and exposed CD-1 males at 20 dpp (D), 12 weeks (E) and 1 year of age (F). Data points represent total SC length from 25–30 pachytene cells from 3–5 CD-1 males. Black bars denote group mean. Placebo and exposure groups were compared by a one-tailed t-test; red asterisk denotes significant difference (p<0.05).

Because recombination levels in mammals are positively correlated with SC length and double strand break (DSB) levels [41–47], we compared RAD51 foci and total SC length between placebo and EE-exposed CD-1 males. To determine if exposure affects the earliest steps in the recombination pathway, we used RAD51 as a surrogate for DSBs, counting the number of foci in zygotene cells (Fig. 4A). No significant differences were observed with EE exposure. Mean RAD51 foci counts were 180.51 ± 3.10 for placebo and 178.41 ± 2.62 for EE-exposed at 20 dpp, and 186.19 ± 3.20 for placebo and 180.43 ± 2.87 for EE-exposed males at 12 weeks (Fig. 4B and C). Total SC lengths were measured in pachytene cells analyzed for MLH1. Although a significant reduction in SC length was observed in EE-exposed males at 20 dpp, surprisingly, no differences between placebo and exposed males were evident in 12 week or 1 year-old males (Fig. 4D–F). Mean SC lengths were 151.08 ± 1.15 for placebo and 140.53 ± 0.95 μm for EE-exposed at 20 dpp (p<0.0001), 152.10 ± 1.06 for placebo and 152.19 ± 0.98 μm for EE-exposed at 12 weeks, and 149.22 ± 1.04 for placebo and 150.37 μm for EE-exposed at 1 year. Further, there was no indication that the relationship between MLH1 levels and SC length was disrupted by either BPA or EE exposure, as a significant positive correlation was observed for each exposure group (S2 Fig.).

## Discussion

Our studies demonstrate that, in the mouse, exposure to exogenous estrogens during testis development permanently alters spermatogenesis in the adult. The nature of the alteration—a reduction in meiotic recombination as assessed by MLH1 levels at pachytene—is interesting for two reasons. First, reducing levels of recombination leads to the failure of some homologs to form a site of exchange, resulting in the robust elimination of spermatocytes [39, 48, 49]. Thus, the reduced recombination evident as both a decrease in MLH1 foci at pachytene and an increased frequency of univalents at MI in exposed males provides mechanistic support for an important tenet of the Estrogen Hypothesis, i.e., that exposure to exogenous estrogens can lead to a reduction in sperm counts. Second, the fact that the recombination phenotype persists when germ cells are transplanted to an unexposed testicular environment suggests that estrogenic exposures induce permanent changes to the SSCs of the developing testis. Importantly, the finding that changes induced in SSCs during testis development influence recombination levels in the adult provides new insight to the setting of meiotic recombination levels in mammals.

### Neonatal estrogenic exposure increases spermatocyte abnormalities

The negative effects of estrogenic chemicals on the developing male include an expanding list of subtle changes to the developing brain, reproductive tract, and testis. Changes in all three have the potential to induce major reproductive repercussions and, although it is widely accepted that developmental exposure adversely impacts spermatogenesis in adulthood, the biological underpinnings remain unclear. Our findings provide a direct mechanistic link between exposure and negative impacts on the germ cell. We observed a reduction in MLH1 levels with estrogenic exposure and a corresponding increase in apparent recombination failure. Consistent with the expectation that recombination failure would lead to an increased incidence of univalents at metaphase I, the incidence of both autosomal and sex chromosome univalents at metaphase I was increased in EE-exposed males (Table 2). Because the action of the spindle assembly checkpoint (SAC) is robust in the male [39, 49], we did not expect that such cells would complete the first meiotic division. Although our data suggest that, indeed, the vast majority of cells with errors were eliminated at MI, we did observe a few abnormal cells at MII in EE-exposed males, including three cells with separated chromatids, two cells with a single extra chromatid, and one cell missing a chromatid (Table 3). These were likely the product of univalents that were able to biorient, as in mitotic cells, and escape detection by the checkpoint at MI [50]. Although we did not test for it, the prediction is that the vast majority of such cells should be eliminated by the actions of the SAC at MII, and the incidence of aneuploid sperm should not be elevated or only slightly elevated in EE-exposed males.

### Evidence that the spermatogonial stem cell is altered

To determine if the recombination effects induced by exogenous estrogens are due to changes in the germline, we transplanted germ cells purified from 13 dpp placebo and EE-exposed males into the testes of W/W^v^ males (Fig. 3). We reasoned that an effect mediated by changes to the somatic cell compartment would be ameliorated by transplantation of germ cells to an unexposed somatic environment. Our meiotic analyses of cells within spermatogenic colonies regenerated by transplanted SSCs, however, demonstrated consistently lower MLH1 levels in the testis transplanted with exposed cells by comparison with the contralateral placebo transplanted testis (Table 4). The persistence of the recombination phenotype in transplanted germ cells provides evidence that estrogenic exposure alters the SSCs. Interestingly, MLH1 levels in meiocytes from both placebo- and EE-exposed males were slightly higher in transplanted testes than in intact C3H males (e.g., 22.98 ± 0.13 placebo vs. 24.11 ± 0.21 transplant placebo, p<0.0001, Table 1 and Table 4), suggesting that the transplantation procedure itself, (e.g., cell isolation, colonization, or genetic differences between donor and recipient) may affect the epigenome of the SSC. Epigenetic changes induced by transplantation may also provide an explanation for the single transplanted male that did not exhibit a recombination difference between the testes transplanted with placebo and exposed cells (Table 4). Nevertheless, the finding of a slight transplantation effect supports the hypothesis that subtle epigenetic changes to the SSC can induce significant changes in recombination.

Estrogen receptors have been reported to be present in spermatogonia in the neonatal testis [51], but whether they are present in the SSC subpopulation remains unclear. It is tempting to conclude that exogenous estrogens exert a direct effect on the SSC population, but the design of our study does not allow us to rule out an indirect effect mediated via changes to the soma. Thus, in future studies, it will be important to determine if estrogen acts directly on the SSCs, e.g., by testing the effect of cell-specific estrogen receptor knockouts.

### Recombination regulation: A new outlook

Although it is clear that the number and placement of the sites of recombination is influenced by both genetic [52–57] and environmental factors [58], when and how recombination levels are set has not been determined in any species. Our current understanding of the control of recombination suggests that MLH1-dependent sites of recombination are influenced by events at two temporally different stages of meiosis—at the time of DSBs formation and Holliday junction resolution. In both mice and humans, levels of recombination have been correlated with both DSB number and SC length [41–47], suggesting that overall levels of recombination are established at or before the onset of DSBs. Based on our previous studies of mouse strains exhibiting significant differences in recombination [47], we anticipated a corresponding 20% reduction in RAD51 foci and 5.5% reduction in SC length. Surprisingly, however, the changes in recombination elicited in response to estrogenic exposure were not accompanied by the expected reduction in either DSBs or SC length. Although we did not test for it, exposure may affect downstream processes involving the proteins, RNF212, HEI10, and CNTD1 that direct recombination site precursors to mature into crossover or noncrossover sites [52, 59–63]. However, it is not immediately obvious why or how alterations to the SSC population of the testis would induce changes in this aspect of DSB repair.

Although the mechanism by which developmental estrogenic exposure acts to alter crossover levels in the adult testis remains to be determined, the finding that recombination changes are linked to the SSC population adds a new layer of complexity, providing the first evidence that meiotic recombination can be affected by events many cell divisions upstream of meiosis. In rodents, the SSC pool is established during neonatal testis development [64]. For the lifespan of the male, the SSCs mitotically divide to regenerate a stem cell and seed a population of cells that undergo successive mitotic divisions before initiating meiosis [65]. Based on our findings, we postulate that estrogenic exposures act to alter the SSC epigenome, and that the altered epigenetic state of the stem cell results in a reduction in crossover levels in downstream meiocyte progeny. Regardless of the mechanism, our findings raise the tantalizing suggestion that in the male, the epigenome of the SSC is key. Importantly, from the standpoint of the reproductive health of humans and other species, they also provide sobering evidence that brief exposures during testis development can have significant and permanent effects on male reproduction.

The consistency of the effect on recombination in exposed males on both the outbred CD-1 and inbred C3H background is striking and suggests that estrogens induce genome-wide epigenetic changes. Notably, our exposure window coincides with the end of the epigenetic reprogramming period in the male germline [66], raising the possibility that estrogenic exposure disrupts remethylation of the SSC genome. If this is indeed the case, the period of vulnerability during which estrogens can act to affect the SSC may be extensive; global demethylation of the germline occurs during migration and after colonization of the testis [67] and reprogramming follows immediately, with an extensive period of DNA remethylation that, in the mouse, extends from approximately 14.5 dpc to several days after birth [68–70]. Thus, to understand the risk posed by estrogenic exposure, it will be important in future studies to carefully delineate the developmental window during which the testis is vulnerable. In this regard, the fact that the B6 strain is not sensitive to the recombination effects of estrogenic exposure makes it a useful tool not only in understanding the epigenetic changes to the SSC that mediate the effect, but also in identifying individuals who will be most susceptible to the effects of environmental estrogens.

### Sex-specific differences in meiotic effects

We previously reported that, in female mice, BPA exposure during fetal development results in an increase in synaptic defects and in MLH1 foci in pachytene stage oocytes [33]. Although we attempted to recapitulate in males the exposure window that induced meiotic effects in the female, the sex-specific differences are striking: In males, synaptic defects are not increased, and meiotic recombination levels are significantly reduced, not increased by exposure. The mechanism of action also appears to be sex-specific. In females, BPA acts as an ER-beta antagonist, phenocopying the absence of ER-beta signaling [33]. In contrast, in the male, the effect is not confined to BPA and EE elicits a more severe reduction in recombination (Table 1), suggesting that meiotic effects in the male may be elicited by a variety of environmental estrogens. Importantly, however, in both sexes exposure can impact the entire reproductive lifespan of the individual, albeit for different reasons: All oocytes enter meiosis prenatally, and exposure coinciding with this period of germ cell development can impact the entire cohort of eggs, reducing the genetic quality of the eggs produced. In males, the current results suggest that perinatal exposure can induce permanent changes to the stem cell population of the testis, permanently changing recombination rates and reducing the number of cells that can successfully complete meiosis and give rise to viable sperm.

In the male, previous studies have reported epigenetic transgenerational effects as a result of exposure to endocrine disruptors [71, 72]. These effects, however, remain poorly understood and extensive analyses of the cells responsible for transmitting effects to subsequent generations—the male germ cells—have not been conducted. Because recombination is a quantitative trait that has been well characterized in mice [47, 73, 74], it provides a sensitive means of tracing effects of exposures across generations and, unlike retrospective molecular analyses of epigenetic changes, allows direct analyses of male germ cells. Thus, in future studies, it will be important to determine if exposure-induced changes in recombination are transmitted to subsequent generations, to define the window of vulnerability of the SSC, and determine if it is limited to the period of neonatal development.

## Materials and Methods

### Animals

Breeding stocks of wildtype male and female ICR (CD-1) mice (Harlan Laboratories, Livermore, CA), C57BL/6J (B6), and C3H/HeJ (C3H) (Jackson Laboratory, Bar Harbor, ME) were maintained in our laboratory in a pathogen-free facility. C3H/B6 F1 hybrids were generated by mating several C3H/HeJ females to a single C57BL/6J male. W/W^v^ males used as germ cell transplantation recipients were generated from breeding heterozygous W and W^v^ C57BL/6J mice (Jackson Laboratory, Bar Harbor, ME). Animals were housed in polysulfone cages (Allentown Inc. Allentown, NJ, Jag 75 micro isolator model) on ventilated racks, in cages containing Sanichip 7090A bedding (Harlan Laboratories) and enrichment material—a nestlet (Ancare, Bellmore, NY), and a Diamond Twist enrichment product (Harlan Laboratories). Drinking water and chow (Purina Lab Diet, 5K52) were provided ad libitum. Individually-housed male animals received a single pellet of Teklad Global 19% Protein Extruded Rodent Diet once a week as an enrichment product (Harlan Laboratories).

### Exposures

Male mice were exposed to 20 or 500 ng/g/day BPA (supplied by NIEHS), 0.25 ng/g/day ethinyl estradiol (Sigma-Aldrich, E4876), or equal volume ethanol/corn oil placebo daily from 1–12 dpp. Chemicals were dissolved in ethanol, diluted in tocopherol-stripped corn oil (MP Biomedicals, Solon, OH), and administered orally by pipette. An exposure level of 20 ng/g/day BPA is below the tolerable daily intake for human consumption (50 ng/g/day) established by the US Environmental Protection Agency and European Food Safety Agency. The 500 ng/g/day dose was chosen as a relevant exposure to humans—it is only slightly higher than 400 ng/g/day, a level that recapitulates blood levels in mice and monkeys similar to blood levels observed in humans [75]. Ethinyl estradiol was chosen as a suitable positive control for oral exposure, and 0.25 ng/g/day EE is well below the recommended levels for comparison with the effects of BPA [22]. To our knowledge, no adverse effects on the testis have been reported for 0.25 ng/g/day EE exposure from 1–12 dpp. For each strain, daily dose was based on the average male pup body weight (g) for each day from 1–12 dpp. A total of six to twelve males (one to three males per litter from at least three litters) were analyzed for each exposure group. Male littermates not utilized for 20 days post-partum (dpp) analyses were weaned and saved for later age analyses at 12 weeks or 1 year of age (CD-1 only). All mouse experiments were approved by the Institutional Animal Care and Use Committee (IACUC) at Washington State University, which is fully accredited by the American Association for Accreditation of Laboratory Animal Care.

### Spermatocyte preparations

Males were killed and testes immediately removed. Spermatocyte preparations were made according to the Peters *et al*. protocol [76] with one modification: a thin layer of 1% paraformaldehyde was applied to clean slides using a glass pipette, rather than by dipping the slide. After overnight incubation in a humid chamber, slides were dried, washed with 0.4% Photo-flo 200 solution (Kodak Professional), air-dried, and viewed on a Nikon Labophot-2 phase microscope. Two slides with spread cells were chosen for immediate staining.

### Immunostaining

Slides were blocked for one hr in sterile filtered antibody dilution buffer (ADB), consisting of 10 ml normal donkey serum (Jackson Immunoresearch), 3 g OmniPur BSA, Fraction V (EMD Millipore), 50 μl Triton X-100 (Alfa Aesar) and 990 ml 1X PBS. For mouse spermatocytes, MLH1 (Calbiochem, PC56, at 1:60) and RAD51 (Santa Cruz biotechnology, sc-8349, at 1:60) primary antibodies were diluted in ADB and 60 μl of antibody solution was applied and covered with 24× 50 mm^2^ glass coverslip, sealed with rubber cement, and incubated overnight at 37°C. Following incubation, coverslips were soaked off in ADB, SYCP3 primary antibody (Santa Cruz biotechnology, sc-74569, at 1:300) with a parafilm coverslip was applied, and slides were incubated for 2 hrs at 37°C. Following incubation, slides were washed in two changes of ADB for at least 1 hr each. Alexa Fluor 488-conjugated AffiniPure Donkey Anti-Rabbit (AFDAR) secondary antibody (Jackson Immunoresearch Laboratories, Inc., 711–545–152, at 1:60) was applied to slides, a glass coverslip added, sealed with rubber cement, and slides were incubated overnight at 37°. The next morning, slides were briefly washed in ADB, and Cy3-conjugated AffiniPure Donkey Anti-Mouse (CDAM) secondary antibody (Jackson Immunoresearch Laboratories, Inc., 715–165–150, at 1:1000) was applied with parafilm coverslip for 45 mins at 37°. At the end of incubation, slides were washed in two changes of 1X PBS for at least 1 hr each, and 20 μl of Prolong Gold antifade reagent with DAPI (Life Technologies, P36931) and glass coverslips were applied. Excess DAPI was blotted out with filter paper, and coverslip edges were sealed with rubber cement. Stained slides were stored at 4° in slide folders prior to analysis.

### MLH1, RAD51, and synaptonemal complex length analyses

Images of cells were captured on a Zeiss Axio Imager epifluorescence microscope. Three images were taken consecutively, SYCP3- TRITC, MLH1 or RAD51- FITC, and DAPI, and cell coordinates were recorded via England finder to allow relocation. Each image was adjusted for uniformity using the Zeiss Axiovision software to reduce background, then saved without the DAPI channel for analysis. MLH1 foci counts were determined for 25–30 pachytene stage cells per animal by two scorers who were blinded with regard to exposure of the animal. Minor scoring discrepancies were resolved and cells with major discrepancies were discarded. Cells with poor staining or synaptic defects were excluded from MLH1 foci number analysis. To assess effects on the formation of the synaptonemal complex (SC), SC lengths were measured from pachytene stage cells used in MLH1 analyses. The total SC length per cell (μm) was obtained using Zeiss Axiovision measuring tools to measure the length of each of the 19 autosomal SCs. The sex chromosome bivalent was excluded from SC length and MLH1 foci analysis. RAD51 foci counts were determined for 20–25 zygotene stage cells per animal and both scores were averaged.

### Synaptic defects

Defects in synapsis were analyzed in 50 pachytene cells from each male. Pachytene cells were selected on the basis of SYCP3 staining, and cells were scored into one of four catagories by two independent observers who were blinded with regard to exposure group. Cells were scored as: 1) perfect, if all homologs were fully synapsed and the sex chromosomes were closely associated, 2) major defects (complete asynapsis, partial asynapsis, non-homologous synapsis), 3) minor defects (forks/bubbles/gaps or fragmentation in an otherwise normal pachytene cell), and 4) associations (nonhomologous end-to-end associations between two or more SCs). Defects were defined as follows—complete asynapsis: one or two pairs of homologs remaining completely unsynapsed. Partial asynapsis: one or two pairs of homologs remaining unsynapsed for at least 1/3 the length of the SC, in a cell that otherwise exhibited complete synapsis. Nonhomologous synapsis: synapsis occurring between nonhomologous chromosomes. Forks and bubbles: one or two pairs of homologs remaining unsynapsed at the end (fork) or interstitially on the SC (bubble) for less than 1/3 of the SC length. Gaps: one or two gaps in SC staining that were longer than the width of an SC. Fragmentation: small segments of SC with colocalized DAPI staining that could not be identified as part of a pair of homologs. Cells with multiple defects were scored in more than one defect category.

### Air-dried preparations

Chromosome preparations were made using the air-dried method of Evans et al. [77]. The frequency of abnormal MI and MII cells was analyzed by two independent observers blinded with respect to exposure group for five EE-exposed and three placebo adult C3H males included in MLH1 analyses. The frequency of univalents was determined for 20–27 MI cells per male, and the frequency of aneuploidy and the presence of monad chromosomes was determined for 20–25 MII cells per male.

### Embryo transfer

Four to five week-old C57BL6/J females were injected with 5 IU pregnant mare serum gonadotropin (National Hormone Peptide Program, Torrance, CA) followed 48 hours later by an injection of 5 IU human chorionic gonadotropin (hCG) (National Hormone Peptide Program, Torrance, CA). Following hCG injection, females were placed overnight with a C57BL/6J male of proven fertility, and checked the following morning for the presence of a vaginal plug. Mated females were euthanized, ovaries and oviducts placed in 1 ml of prewarmed M2 medium (Millipore MR-015P) in a 60 mm tissue culture dish, and cumulus-covered one-cell embryos were released by carefully tearing the ampulla open with fine forceps. Hyaluronidase solution (0.3mg/ml, Sigma H3506) was added to medium to free adherent cumulus cells. One-cell embryos were collected via glass transfer pipette, washed in three separate drops of medium, moved to a drop of medium covered in oil, and incubated at 37°C with 5% CO_2_ for 10–30 minutes before transfer. Pseudopregnant (0.5 days) CD-1 or C57BL/6J females were obtained from matings with vasectomized CD-1 males. Approximately 10 one-cell embryos were transferred via glass pipette to the infundibulum of each oviduct of anesthetized pseudopregnant females.

### Germ cell transplantation

Testes of 0.25 ng/g EE or placebo C3H males were collected in HBSS at the end of the exposure period (13 dpp). Cell suspensions were prepared using the digestion and percoll selection steps as described previously [78]. Cells were suspended at a concentration of 3 × 10^6^ cells/ml in cold dPBSS and kept on ice until transplantation. Approximately 5–10 μl of placebo cell suspension was injected into seminiferous tubules via the efferent ducts of one testis of the adult W/W^v^ recipient [78]. EE-exposed cells were injected into the contralateral testis. Exposure groups alternated between the left and right testis to account for any differences between testes or timing (first vs. second injection). Mice were kept for a minimum of eight weeks post transplantation to allow colonization and multiple cycles of spermatogenesis before meiotic analyses. All W/W^v^ recipients were analyzed prior to six months of age. Because colonies occur in localized patches, the entire testis was used for surface spread preparations to ensure enough cells were obtained. Each W/W^v^ testis was divided into five pieces, each piece providing the material for two slides.

### Statistical analysis

Among-group differences in mean MLH1 foci were analyzed by one-way ANOVA for CD-1 and B6 exposure groups, and embryo transfer groups. For statistically significant differences (p<0.05), a Newman-Keuls post hoc test was performed to infer which groups differed. An unpaired t-test was used in instances where only two groups were being tested, including MLH1 analyses for C3H, C3H/B6 F1, and germ cell transplantation analyses, RAD51, and SC lengths. Chi-square analyses were used to determine differences in the frequency of cells containing SCs without recombination sites and abnormal cells at MI.

## Supporting Information

S1 Table Analysis of synaptic defects in pachytene cells with neonatal estrogenic exposure. Values represent percentage of cells.
(DOCX)

S2 Table Recombination rate in B6 males transferred as one-cell embryos to CD-1 females and orally exposed to ethinyl estradiol after birth. *n = number of cells analyzed at 20 dpp. ^a, b^Groups were compared by one-way ANOVA. Letters denote significant differences as determined by a Newman-Keuls post hoc test (at least p<0.05); like letters indicate no difference.
(DOCX)

S3 Table Distribution of MLH1 sites in placebo and exposed males. *Values represent percentage of SCs with 0, 1, 2, or 3 MLH1 foci in pachytene cells.
(DOCX)

S1 Fig Recombination rates in male mice orally exposed to BPA or ethinyl estradiol. Data points represent mean MLH1 counts for individual males; bars represent group mean.
(PDF)

S2 Fig Relationship between recombination and synaptonemal complex length is unperturbed by exposure in CD-1 males. Data points represent total MLH1 foci (x-axis) and corresponding SC length (y-axis) for pachytene cells in 20 dpp, 12 week, and 1 year-old CD-1 males neonatally exposed to EE from 1–12 dpp. Pearson correlation coefficients were calculated to determine relationship between recombination and synaptonemal complex length. For CD-1, the Pearson correlation coefficients were 0.57 (p<0.0001) for placebo and 0.36 (p<0.0001) for 0.25 ng EE-exposed males at 20 dpp and 0.37 (p<0.0001) for placebo and 0.34 (p<0.0001) for 0.25 ng EE-exposed males at 12 weeks old, and 0.35 (p<0.0001) for placebo and 0.48 (p<0.0001) for 0.25 ng EE-exposed males at 1 year old.
(PDF)

## References

[1] Carlsen E, Giwercman A, Keiding N, Skakkebaek NE (1992) Evidence for decreasing quality of semen during past 50 years. BMJ 305: 609–613. 10.1136/bmj.305.6854.609 1393072 PMC1883354

[2] Auger J, Kunstmann JM, Czyglik F, Jouannet P (1995) Decline in semen quality among fertile men in Paris during the past 20 years. N Engl J Med 332: 281–285. 10.1056/NEJM199502023320501 7816062

[3] Bonde JP, Kold Jensen T, Brixen Larsen S, Abell A, Scheike T, et al (1998) Year of birth and sperm count in 10 Danish occupational studies. Scand J Work Environ Health 24: 407–413. 10.5271/sjweh.362 9869313

[4] Andersen AG (2000) High frequency of sub-optimal semen quality in an unselected population of young men. Hum Reprod 15: 366–372. 10.1093/humrep/15.2.366 10655308

[5] Jørgensen N, Carlsen E, Nermoen I, Punab M, Suominen J, et al (2002) East-West gradient in semen quality in the Nordic-Baltic area: a study of men from the general population in Denmark, Norway, Estonia and Finland. Hum Reprod 17: 2199–2208. 10.1093/humrep/17.8.2199 12151459

[6] Jørgensen N, Joensen UN, Jensen TK, Jensen MB, Almstrup K, et al (2012) Human semen quality in the new millennium: a prospective cross-sectional population-based study of 4867 men. BMJ Open 2 10.1136/bmjopen-2012-000990 22761286 PMC3391374

[7] Iwamoto T, Nozawa S, Mieno MN, Yamakawa K, Baba K, et al (2013) Semen quality of 1559 young men from four cities in Japan: a cross-sectional population-based study. BMJ Open 3: 4–8. 10.1136/bmjopen-2012-002222 23633418 PMC3641477

[8] Jørgensen N, Vierula M, Jacobsen R, Pukkala E, Perheentupa A, et al (2011) Recent adverse trends in semen quality and testis cancer incidence among Finnish men. Int J Androl 34: e37–e48. 10.1111/j.1365-2605.2010.01133.x 21366607 PMC3170483

[9] Mendiola J, Jørgensen N, Mínguez-Alarcón L, Sarabia-Cos L, López-Espín JJ, et al (2013) Sperm counts may have declined in young university students in Southern Spain. Andrology 1: 408–413. 10.1111/j.2047-2927.2012.00058.x 23307495

[10] Mendiola J, Jørgensen N, Andersson A-M, Stahlhut RW, Liu F, et al (2014) Reproductive parameters in young men living in Rochester, New York. Fertil Steril 101: 1064–1071. 10.1016/j.fertnstert.2014.01.007 24524829

[11] Rolland M, Le Moal J, Wagner V, Royère D, De Mouzon J (2013) Decline in semen concentration and morphology in a sample of 26,609 men close to general population between 1989 and 2005 in France. Hum Reprod 28: 462–470. 10.1093/humrep/des415 23213178 PMC4042534

[12] Sharpe RM (2003) The “oestrogen hypothesis” – where do we stand now ? 1 15: 2–15. 10.1046/j.1365-2605.2003.00367.x 12534932

[13] Sharpe RM, Skakkebaek NE (1993) Are oestrogens involved in falling sperm counts and disorders of the male reproductive tract? Lancet 341: 1392–1395. 10.1016/0140-6736(93)90953-E 8098802

[14] Cosgrove M., Benton B, Henderson BE (1977) Coscrove et al 1977.pdf. J Urol. 875202

[15] Wilcox AJ, Baird DD, Weinberg CR, Hornsby PP, Herbst AL (1995) Fertility in Men Exposed Prenatally to Diethylstilbestrol—NEJM. NEJM. 10.1056/NEJM199505253322104 7723797

[16] Strohsnitter WC, Noller KL, Hoover RN, Robboy SJ, Palmer JR, et al (2001) Cancer Risk in Men Exposed In Utero to Diethylstilbestrol. JNCI J Natl Cancer Inst 93: 545–551. 10.1093/jnci/93.7.545 11287449

[17] Palmer JR, Herbst AL, Noller KL, Boggs DA, Troisi R, et al (2009) Urogenital abnormalities in men exposed to diethylstilbestrol in utero: a cohort study. Environ Health 8: 37 10.1186/1476-069X-8-37 19689815 PMC2739506

[18] Gill WB, Schumacher FB, Bibbo M, Straus FH, Schoenberg HW (1979) Gill et al 1979.pdf. J Urol. 10.1016/s0022-5347(17)56240-0 37351

[19] Atanassova N, McKinnell C, Walker M, Turner KJ, Fisher JS, et al (1999) Permanent effects of neonatal estrogen exposure in rats on reproductive hormone levels, Sertoli cell number, and the efficiency of spermatogenesis in adulthood. Endocrinology 140: 5364–5373. 10.1210/endo.140.11.7108 10537168

[20] Vom Saal FS, Cooke PS, Buchanan DL, Palanza P, Thayer KA, et al (1998) A physiologically based approach to the study of bisphenol A and other estrogenic chemicals on the size of reproductive organs, daily sperm production, and behavior. Toxicol Ind Health 14: 239–260. 10.1177/074823379801400115 9460178

[21] Thayer KA, Ruhlen RL, Howdeshell KL, Buchanan DL, Cooke PS, et al (2001) Altered prostate growth and daily sperm production in male mice exposed prenatally to subclinical doses of 17alpha-ethinyl oestradiol. Hum Reprod 16: 988–996. 10.1093/humrep/16.5.988 11331650

[22] Richter CA, Birnbaum LS, Farabollini F, Newbold RR, Rubin BS, et al (2007) In vivo effects of bisphenol A in laboratory rodent studies. Reprod Toxicol 24: 199–224. 10.1016/j.reprotox.2007.06.004 17683900 PMC2151845

[23] Howdeshell KL, Furr J, Lambright CR, Wilson VS, Ryan BC, et al (2008) Gestational and lactational exposure to ethinyl estradiol, but not bisphenol A, decreases androgen-dependent reproductive organ weights and epididymal sperm abundance in the male long evans hooded rat. Toxicol Sci 102: 371–382. 10.1093/toxsci/kfm306 18096570

[24] Frye CA, Bo E, Calamandrei G, Calzà L, Dessì-Fulgheri F, et al (2012) Endocrine disrupters: a review of some sources, effects, and mechanisms of actions on behaviour and neuroendocrine systems. J Neuroendocrinol 24: 144–159. 10.1111/j.1365-2826.2011.02229.x 21951193 PMC3245362

[25] Atanassova N, McKinnell C, Fisher J, Sharpe RM (2005) Neonatal treatment of rats with diethylstilboestrol (DES) induces stromal-epithelial abnormalities of the vas deferens and cauda epididymis in adulthood following delayed basal cell development. Reproduction 129: 589–601. 10.1530/rep.1.00546 15855622

[26] Mathews E, Braden TD, Williams CS, Williams JW, Bolden-Tiller O, et al (2009) Mal-development of the penis and loss of fertility in male rats treated neonatally with female contraceptive 17alpha-ethinyl estradiol: a dose-response study and a comparative study with a known estrogenic teratogen diethylstilbestrol. Toxicol Sci 112: 331–343. 10.1093/toxsci/kfp207 19729556 PMC2777077

[27] Akingbemi BT, Sottas CM, Koulova AI, Klinefelter GR, Hardy MP (2004) Inhibition of testicular steroidogenesis by the xenoestrogen bisphenol A is associated with reduced pituitary luteinizing hormone secretion and decreased steroidogenic enzyme gene expression in rat Leydig cells. Endocrinology 145: 592–603. 10.1210/en.2003-1174 14605012

[28] N’Tumba-Byn T, Moison D, Lacroix M, Lecureuil C, Lesage L, et al (2012) Differential effects of bisphenol A and diethylstilbestrol on human, rat and mouse fetal leydig cell function. PLoS One 7: e51579 10.1371/journal.pone.0051579 23284716 PMC3524173

[29] Sharpe RM, Rivas A, Walker M, McKinnell C, Fisher JS (2003) Effect of neonatal treatment of rats with potent or weak (environmental) oestrogens, or with a GnRH antagonist, on Leydig cell development and function through puberty into adulthood. Int J Androl 26: 26–36. 10.1046/j.1365-2605.2003.00385.x 12534935

[30] Koh K-B, Toyama Y, Komiyama M, Adachi T, Fukata H, et al (2006) Neonatal administration of diethylstilbestrol has adverse effects on somatic cells rather than germ cells. Reprod Toxicol 22: 746–753. 10.1016/j.reprotox.2006.07.006 17005366

[31] Salian S, Doshi T, Vanage G (2009) Neonatal exposure of male rats to Bisphenol A impairs fertility and expression of sertoli cell junctional proteins in the testis. Toxicology 265: 56–67. 10.1016/j.tox.2009.09.012 19782717

[32] Hunt PA, Koehler KE, Susiarjo M, Hodges CA, Ilagan A, et al (2003) Bisphenol a exposure causes meiotic aneuploidy in the female mouse. Curr Biol 13: 546–553. 10.1016/S0960-9822(03)00189-1 12676084

[33] Susiarjo M, Hassold TJ, Freeman E, Hunt PA (2007) Bisphenol A exposure in utero disrupts early oogenesis in the mouse. PLoS Genet 3: e5 10.1371/journal.pgen.0030005 17222059 PMC1781485

[34] Caldwell DJ, Mastrocco F, Nowak E, Johnston J, Yekel H, et al (2010) An assessment of potential exposure and risk from estrogens in drinking water. Environ Health Perspect 118: 338–344. 10.1289/ehp.0900654 20194073 PMC2854760

[35] Wise A, O’Brien K, Woodruff T (2011) Are oral contraceptives a significant contributor to the estrogenicity of drinking water? Environ Sci Technol 45: 51–60. 10.1021/es1014482 20977246

[36] Spearow JL, Doemeny P, Sera R, Leffler R, Barkley M (1999) Genetic variation in susceptibility to endocrine disruption by estrogen in mice. Science 285: 1259–1261. 10.1126/science.285.5431.1259 10455051

[37] Svetlanov A, Baudat F, Cohen PE, de Massy B (2008) Distinct functions of MLH3 at recombination hot spots in the mouse. Genetics 178: 1937–1945. 10.1534/genetics.107.084798 18430927 PMC2323788

[38] Svare B, Kinsley CH, Mann MA, Broida J (1984) Svare et al.pdf. Physiol Behav 33: 137–152. 10.1016/0031-9384(84)90024-6 6542232

[39] Sutcliffe MJ, Darling SM, Burgoyne PS (1991) Spermatogenesis in XY, XYSxra and XOSxra mice: a quantitative analysis of spermatogenesis throughout puberty. Mol Reprod Dev 30: 81–89. 10.1002/mrd.1080300202 1954032

[40] McLean DJ (2008) Spermatogonial stem cell transplantation, testicular function, and restoration of male fertility in mice. Methods Mol Biol 450: 149–162. 10.1007/978-1-60327-214-8_11 18370058

[41] Barlow AL, Benson FE, West SC, Hultén MA (1997) Distribution of the Rad51 recombinase in human and mouse spermatocytes. EMBO J 16: 5207–5215. 10.1093/emboj/16.17.5207 9311981 PMC1170153

[42] Lynn A, Koehler KE, Judis L, Chan ER, Cherry JP, et al (2002) Covariation of synaptonemal complex length and mammalian meiotic exchange rates. Science 296: 2222–2225. 10.1126/science.1071220 12052900

[43] Tease C, Hultén MA (2004) Inter-sex variation in synaptonemal complex lengths largely determine the different recombination rates in male and female germ cells. Cytogenet Genome Res 107: 208–215. 10.1159/000080599 15467366

[44] Lenzi ML, Smith J, Snowden T, Kim M, Fishel R, et al (2005) Extreme heterogeneity in the molecular events leading to the establishment of chiasmata during meiosis i in human oocytes. Am J Hum Genet 76: 112–127. 10.1086/427268 15558497 PMC1196414

[45] Oliver-Bonet M, Turek PJ, Sun F, Ko E, Martin RH (2005) Temporal progression of recombination in human males. Mol Hum Reprod 11: 517–522. 10.1093/molehr/gah193 16123081

[46] Gruhn JR, Rubio C, Broman KW, Hunt PA, Hassold T (2013) Cytological studies of human meiosis: sex-specific differences in recombination originate at, or prior to, establishment of double-strand breaks. PLoS One 8: e85075 10.1371/journal.pone.0085075 24376867 PMC3869931

[47] Baier B, Hunt P, Broman KW, Hassold T (2014) Variation in Genome-Wide Levels of Meiotic Recombination Is Established at the Onset of Prophase in Mammalian Males. PLoS Genet 10: e1004125 10.1371/journal.pgen.1004125 24497841 PMC3907295

[48] Eaker S, Cobb J, Pyle A, Handel MA (2002) Meiotic prophase abnormalities and metaphase cell death in MLH1-deficient mouse spermatocytes: insights into regulation of spermatogenic progress. Dev Biol 249: 85–95. 10.1006/dbio.2002.0708 12217320

[49] Vernet N, Mahadevaiah SK, Ojarikre OA, Longepied G, Prosser HM, et al (2011) The Y-encoded gene zfy2 acts to remove cells with unpaired chromosomes at the first meiotic metaphase in male mice. Curr Biol 21: 787–793. 10.1016/j.cub.2011.03.057 21530259 PMC3176893

[50] Kouznetsova A, Lister L, Nordenskjöld M, Herbert M, Höög C (2007) Bi-orientation of achiasmatic chromosomes in meiosis I oocytes contributes to aneuploidy in mice. Nat Genet 39: 966–968. 10.1038/ng2065 17618286

[51] Jefferson WN, Couse JF, Banks EP, Korach KS, Newbold RR (2000) Expression of estrogen receptor beta is developmentally regulated in reproductive tissues of male and female mice. Biol Reprod 62: 310–317. 10.1095/biolreprod62.2.310 10642567

[52] Kong A, Thorleifsson G, Stefansson H, Masson G, Helgason A, et al (2008) Sequence variants in the RNF212 gene associate with genome-wide recombination rate. Science 319: 1398–1401. 10.1126/science.1152422 18239089

[53] Chowdhury R, Bois PRJ, Feingold E, Sherman SL, Cheung VG (2009) Genetic analysis of variation in human meiotic recombination. PLoS Genet 5: e1000648 10.1371/journal.pgen.1000648 19763160 PMC2730532

[54] Baudat F, Buard J, Grey C, Fledel-Alon A, Ober C, et al (2010) PRDM9 is a major determinant of meiotic recombination hotspots in humans and mice. Science 327: 836–840. 10.1126/science.1183439 20044539 PMC4295902

[55] Parvanov ED, Petkov PM, Paigen K (2010) Prdm9 controls activation of mammalian recombination hotspots. Science 327: 835 10.1126/science.1181495 20044538 PMC2821451

[56] Murdoch B, Owen N, Shirley S, Crumb S, Broman KW, et al (2010) Multiple loci contribute to genome-wide recombination levels in male mice. Mamm Genome 21: 550–555. 10.1007/s00335-010-9303-5 21113599 PMC3002158

[57] Sandor C, Li W, Coppieters W, Druet T, Charlier C, et al (2012) Genetic variants in REC8, RNF212, and PRDM9 influence male recombination in cattle. PLoS Genet 8: e1002854 10.1371/journal.pgen.1002854 22844258 PMC3406008

[58] Lynn A, Schrump S, Cherry J, Hassold T, Hunt P (2005) Sex, not genotype, determines recombination levels in mice. Am J Hum Genet 77: 670–675. 10.1086/491718 16175513 PMC1275616

[59] Ward JO, Reinholdt LG, Motley WW, Niswander LM, Deacon DC, et al (2007) Mutation in mouse hei10, an e3 ubiquitin ligase, disrupts meiotic crossing over. PLoS Genet 3: e139 10.1371/journal.pgen.0030139 17784788 PMC1959360

[60] Reynolds A, Qiao H, Yang Y, Chen JK, Jackson N, et al (2013) RNF212 is a dosage-sensitive regulator of crossing-over during mammalian meiosis. Nat Genet 45: 269–278. 10.1038/ng.2541 23396135 PMC4245152

[61] Qiao H, Prasada Rao HBD, Yang Y, Fong JH, Cloutier JM, et al (2014) Antagonistic roles of ubiquitin ligase HEI10 and SUMO ligase RNF212 regulate meiotic recombination. Nat Genet 46: 194–199. 10.1038/ng.2858 24390283 PMC4356240

[62] De Muyt A, Zhang L, Piolot T, Kleckner N, Espagne E, et al (2014) E3 ligase Hei10: a multifaceted structure-based signaling molecule with roles within and beyond meiosis. Genes Dev 28: 1111–1123. 10.1101/gad.240408.114 24831702 PMC4035539

[63] Holloway JK, Sun X, Yokoo R, Villeneuve AM, Cohen PE (2014) Mammalian CNTD1 is critical for meiotic crossover maturation and deselection of excess precrossover sites. J Cell Biol 205: 633–641. 10.1083/jcb.201401122 24891606 PMC4050721

[64] Oatley JM, Brinster RL (2012) The germline stem cell niche unit in mammalian testes. Physiol Rev 92: 577–595. 10.1152/physrev.00025.2011 22535892 PMC3970841

[65] Ryu B-Y, Orwig KE, Oatley JM, Avarbock MR, Brinster RL (2006) Effects of aging and niche microenvironment on spermatogonial stem cell self-renewal. Stem Cells 24: 1505–1511. 10.1634/stemcells.2005-0580 16456131 PMC5501308

[66] Trasler JM (2009) Epigenetics in spermatogenesis. Mol Cell Endocrinol 306: 33–36. 10.1016/j.mce.2008.12.018 19481683

[67] Seki Y, Hayashi K, Itoh K, Mizugaki M, Saitou M, et al (2005) Extensive and orderly reprogramming of genome-wide chromatin modifications associated with specification and early development of germ cells in mice. Dev Biol 278: 440–458. 10.1016/j.ydbio.2004.11.025 15680362

[68] Davis TL, Yang GJ, McCarrey JR, Bartolomei MS (2000) The H19 methylation imprint is erased and re-established differentially on the parental alleles during male germ cell development. Hum Mol Genet 9: 2885–2894. 10.1093/hmg/9.19.2885 11092765

[69] Ueda T, Abe K, Miura A, Yuzuriha M, Zubair M, et al (2000) The paternal methylation imprint of the mouse H19 locus is acquired in the gonocyte stage during foetal testis development. Genes Cells 5: 649–659. 10.1046/j.1365-2443.2000.00351.x 10947850

[70] Kato Y, Kaneda M, Hata K, Kumaki K, Hisano M, et al (2007) Role of the Dnmt3 family in de novo methylation of imprinted and repetitive sequences during male germ cell development in the mouse. Hum Mol Genet 16: 2272–2280. 10.1093/hmg/ddm179 17616512

[71] Anway MD, Memon MA, Uzumcu M, Skinner MK (n.d) Transgenerational effect of the endocrine disruptor vinclozolin on male spermatogenesis. J Androl 27: 868–879. 10.2164/jandrol.106.000349 16837734 PMC11451258

[72] Manikkam M, Tracey R, Guerrero-Bosagna C, Skinner MK (2013) Plastics derived endocrine disruptors (BPA, DEHP and DBP) induce epigenetic transgenerational inheritance of obesity, reproductive disease and sperm epimutations. PLoS One 8: e55387 10.1371/journal.pone.0055387 23359474 PMC3554682

[73] Koehler KE, Cherry JP, Lynn A, Hunt PA, Hassold TJ (2002) Genetic control of mammalian meiotic recombination. I. Variation in exchange frequencies among males from inbred mouse strains. Genetics 162: 297–306. 12242241 10.1093/genetics/162.1.297 PMC1462263

[74] Dumont BL, White MA, Steffy B, Wiltshire T, Payseur BA (2011) Extensive recombination rate variation in the house mouse species complex inferred from genetic linkage maps. Genome Res 21: 114–125. 10.1101/gr.111252.110 20978138 PMC3012918

[75] Taylor J a, Vom Saal FS, Welshons W V, Drury B, Rottinghaus G, et al (2011) Similarity of bisphenol A pharmacokinetics in rhesus monkeys and mice: relevance for human exposure. Environ Health Perspect 119: 422–430. 10.1289/ehp.1002514 20855240 PMC3080921

[76] Peters AH, Plug AW, van Vugt MJ, de Boer P (1997) A drying-down technique for the spreading of mammalian meiocytes from the male and female germline. Chromosome Res 5: 66–68. 10.1023/A:1018445520117 9088645

[77] Evans EP, Breckon G, Ford CE (1964) AN AIR-DRYING METHOD FOR MEIOTIC PREPARATIONS FROM MAMMALIAN TESTES. Cytogenetics 3: 289–294. 10.1159/000129818 14248459

[78] Oatley JM, Brinster RL (2006) Spermatogonial stem cells. Methods Enzymol 419: 259–282. 10.1016/S0076-6879(06)19011-4 17141059

