# Supplementary material for: Estrogenic Exposure Alters the Spermatogonial Stem Cells in the Developing Testis, Permanently Reducing Crossover Levels in the Adult
Source: PLoS Genet. 2015 Jan 23;11(1):e1004949. doi: 10.1371/journal.pgen.1004949 (PMC4304829; doi:10.1371/journal.pgen.1004949)
Supplement: S1 Table — Values represent percentage of cells. (DOCX) [file pgen.1004949.s001.docx]

Table S1. Analysis of synaptic defects in pachytene cells with neonatal estrogenic exposure

|  |  | Number of cells | Perfect | Major defects | Minor defects | Associations |
| --- | --- | --- | --- | --- | --- | --- |
| 20 dpp |  |  |  |  |  |  |
| CD-1 | Placebo | 500 | 91.40 | 1.80 | 4.40 | 3.00 |
|  | 20 ng BPA | 600 | 93.67 | 1.00 | 2.67 | 2.50 |
|  | 500 ng BPA | 500 | 92.40 | 1.80 | 4.40 | 1.60 |
|  | 0.25 ng EE | 350 | 91.14 | 1.71 | 3.71 | 3.43 |
|  |  |  |  |  |  |  |
| B6 | Placebo | 300 | 94.66 | 1.67 | 3.33 | 1.00 |
|  | 20 ng BPA | 350 | 91.50 | 5.71 | 4.00 | 3.14 |
|  | 500 ng BPA | 400 | 91.80 | 3.25 | 5.00 | 2.00 |
|  | 0.25 ng EE | 350 | 92.50 | 6.29 | 6.00 | 1.40 |
|  |  |  |  |  |  |  |
| C3H | Placebo | 300 | 93.00 | 1.00 | 4.33 | 3.33 |
|  | 0.25 ng EE | 300 | 90.67 | 4.67 | 3.00 | 4.00 |
|  |  |  |  |  |  |  |
| 12 weeks |  |  |  |  |  |  |
| CD-1 | Placebo | 350 | 91.15 | 1.43 | 6.57 | 2.29 |
|  | 20 ng BPA | 300 | 95.33 | 2.00 | 0.67 | 2.67 |
|  | 500 ng BPA | 300 | 94.33 | 2.33 | 3.67 | 2.33 |
|  | 0.25 ng EE | 300 | 97.67 | 1.67 | 0.33 | 2.33 |
|  |  |  |  |  |  |  |
| B6 | Placebo | 350 | 96.28 | 4.29 | 1.14 | 1.43 |
|  | 20 ng BPA | 3000 | 95.00 | 3.00 | 2.33 | 0.67 |
|  | 500 ng BPA | 250 | 96.00 | 1.60 | 2.40 | 1.20 |
|  | 0.25 ng EE | 250 | 89.60 | 2.40 | 3.20 | 5.60 |
|  |  |  |  |  |  |  |
| C3H | Placebo | 200 | 84.00 | 0.50 | 1.00 | 17.50 |
|  | 0.25 ng EE | 250 | 83.20 | 0.00 | 2.80 | 15.20 |
|  |  |  |  |  |  |  |
| 1 year |  |  |  |  |  |  |
| CD-1 | Placebo | 250 | 95.60 | 4.80 | 1.20 | 4.00 |
|  | 20 ng BPA | 200 | 79.00 | 11.00 | 2.50 | 10.50 |
|  | 500 ng BPA | 200 | 93.00 | 5.50 | 2.50 | 3.50 |
|  | 0.25 ng EE | 200 | 92.00 | 0.50 | 3.50 | 5.00 |

Values represent percentage of cells.
