# Supplementary material for: Estrogenic Exposure Alters the Spermatogonial Stem Cells in the Developing Testis, Permanently Reducing Crossover Levels in the Adult
Source: PLoS Genet. 2015 Jan 23;11(1):e1004949. doi: 10.1371/journal.pgen.1004949 (PMC4304829; doi:10.1371/journal.pgen.1004949)
Supplement: S2 Table — *n = number of cells analyzed at 20 dpp. a, bGroups were compared by one-way ANOVA. Letters denote significant differences as determined by a Newman-Keuls post hoc test (at least p<0.05); like letters indicate no difference. (DOCX) [file pgen.1004949.s002.docx]

Table S2. Recombination rate in B6 males transferred as one-cell embryos to CD-1 females and orally exposed to ethinyl estradiol after birth.

| Embryo/pup genotype | Genotype of pseudopregnant female | Genotype of postpartum mother | Exposure | n* | Mean MLH1 ± SEM |
| --- | --- | --- | --- | --- | --- |
| B6 | CD-1 | CD-1 | Placebo | 229 | 22.30 ± 0.14^a^ |
| B6 | CD-1 | CD-1 | 0.25 ng EE | 167 | 22.32 ± 0.14^a^ |
| B6 | B6 | B6 | Placebo | 140 | 22.93 ± 0.15^b^ |
| B6 |  | CD-1 | Placebo | 253 | 23.23 ± 0.12^b^ |

*n= number of cells analyzed at 20 dpp.

^a,b^Groups were compared by one-way ANOVA. Letters denote significant differences as determined by a Newman-Keuls post hoc test (at least p<0.05); like letters indicate no difference.
