# Supplementary material for: Estrogenic Exposure Alters the Spermatogonial Stem Cells in the Developing Testis, Permanently Reducing Crossover Levels in the Adult
Source: PLoS Genet. 2015 Jan 23;11(1):e1004949. doi: 10.1371/journal.pgen.1004949 (PMC4304829; doi:10.1371/journal.pgen.1004949)
Supplement: S3 Table — *Values represent percentage of SCs with 0, 1, 2, or 3 MLH1 foci in pachytene cells. (DOCX) [file pgen.1004949.s003.docx]

Table S3. Distribution of MLH1 sites in placebo and exposed males.

|  |  | number of SCs | MLH1 foci per SC* | | | |
| --- | --- | --- | --- | --- | --- | --- |
|  |  |  | 0 | 1 | 2 | 3 |
| 20 dpp |  |  |  |  |  |  |
| CD-1 | Placebo | 5833 | 0.17 | 82.70 | 16.89 | 0.24 |
|  | 20 ng BPA | 6422 | 0.12 | 86.76 | 12.94 | 0.17 |
|  | 500 ng BPA | 5511 | 0.18 | 85.25 | 14.33 | 0.24 |
|  | 0.25 ng EE | 3743 | 0.80 | 88.67 | 10.47 | 0.05 |
|  |  |  |  |  |  |  |
| B6 | Placebo | 3458 | 0.38 | 78.80 | 20.47 | 0.35 |
|  | 20 ng BPA | 3800 | 0.29 | 78.84 | 20.58 | 0.29 |
|  | 500 ng BPA | 4265 | 0.16 | 78.55 | 20.82 | 0.47 |
|  | 0.25 ng EE | 3800 | 0.53 | 77.89 | 21.32 | 0.26 |
|  |  |  |  |  |  |  |
| C3H | Placebo | 3268 | 0.24 | 85.34 | 14.23 | 0.18 |
|  | 0.25 ng EE | 2792 | 1.43 | 89.94 | 8.49 | 0.14 |
|  |  |  |  |  |  |  |
| C3H/B6 F1 | Placebo | 1102 | 0.09 | 75.23 | 24.59 | 0.09 |
|  | 0.25 ng EE | 2223 | 0.13 | 78.09 | 21.64 | 0.13 |
|  |  |  |  |  |  |  |
| 12 weeks |  |  |  |  |  |  |
| CD-1 | Placebo | 3667 | 0.25 | 72.05 | 27.38 | 0.33 |
|  | 20 ng BPA | 3249 | 0.55 | 76.67 | 22.71 | 0.06 |
|  | 500 ng BPA | 2830 | 0.35 | 78.73 | 20.74 | 0.18 |
|  | 0.25 ng EE | 3439 | 0.47 | 84.82 | 14.63 | 0.09 |
|  |  |  |  |  |  |  |
| B6 | Placebo | 3610 | 0.58 | 72.77 | 26.32 | 0.33 |
|  | 20 ng BPA | 3240 | 0.65 | 70.90 | 28.21 | 0.25 |
|  | 500 ng BPA | 2698 | 0.59 | 73.80 | 24.87 | 0.74 |
|  | 0.25 ng EE | 2451 | 0.29 | 74.34 | 25.21 | 0.16 |
|  |  |  |  |  |  |  |
| C3H | Placebo | 4883 | 0.10 | 78.95 | 20.77 | 0.18 |
|  | 0.25 ng EE | 5263 | 0.53 | 83.30 | 16.13 | 0.04 |
|  |  |  |  |  |  |  |
| C3H/B6 F1 | Placebo | 4237 | 0.09 | 69.53 | 30.04 | 0.33 |
|  | 0.25 ng EE | 4066 | 0.12 | 71.45 | 28.14 | 0.30 |
|  |  |  |  |  |  |  |
| 1 year |  |  |  |  |  |  |
| CD-1 | Placebo | 2661 | 0.34 | 70.61 | 28.86 | 0.19 |
|  | 20 ng BPA | 2071 | 1.06 | 76.29 | 22.36 | 0.29 |
|  | 500 ng BPA | 2014 | 1.74 | 73.68 | 24.28 | 0.30 |
|  | 0.25 ng EE | 2508 | 0.52 | 72.97 | 26.32 | 0.20 |

*Values represent percentage of SCs with 0, 1, 2, or 3 MLH1 foci in pachytene cells.
