# Supplementary figures and images for: Estrogenic Exposure Alters the Spermatogonial Stem Cells in the Developing Testis, Permanently Reducing Crossover Levels in the Adult
Source: PLoS Genet. 2015 Jan 23;11(1):e1004949. doi: 10.1371/journal.pgen.1004949 (PMC4304829; doi:10.1371/journal.pgen.1004949)

20 dpp

12 weeks

1 year

CD-1

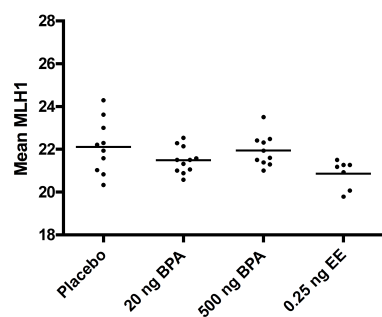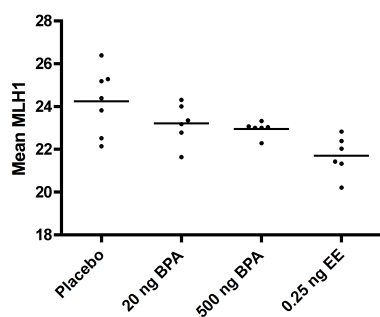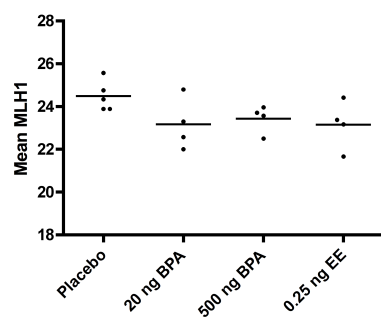

C57BL/6J

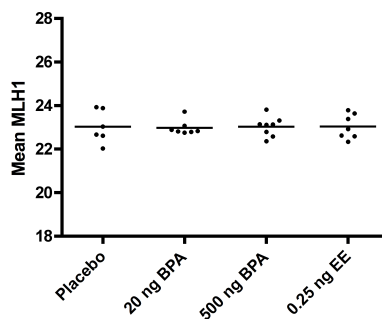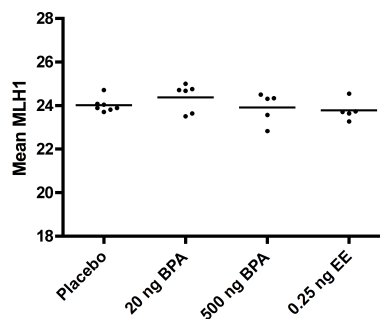

C3H/HeJ

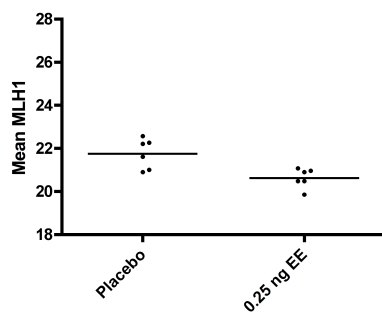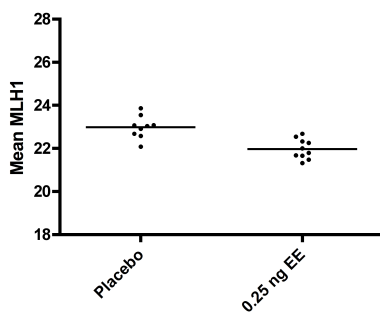

C3HxB6 F1

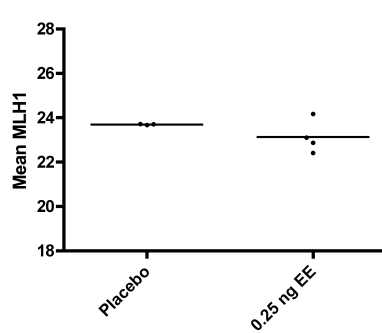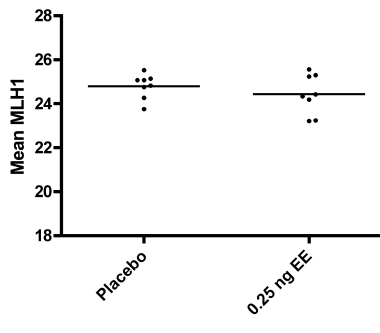

Supplement: S1 Fig — Data points represent mean MLH1 counts for individual males; bars represent group mean. (PDF) [file pgen.1004949.s004.pdf]

Placebo

20 dpp

12 weeks

1 year

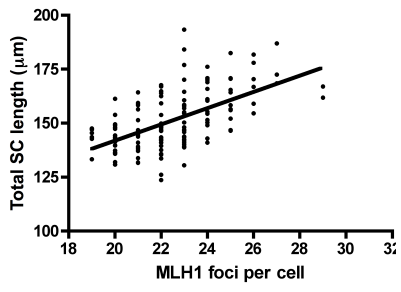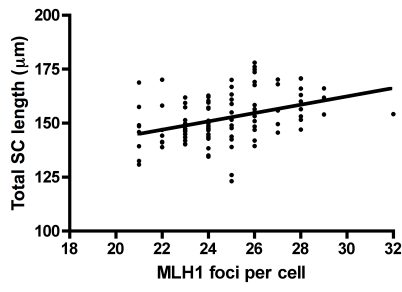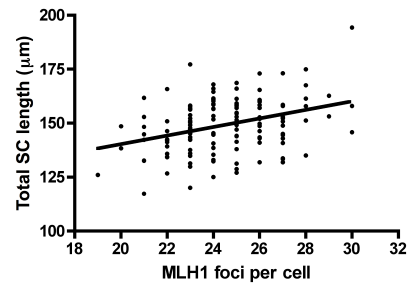

0.25 ng/g/day EE

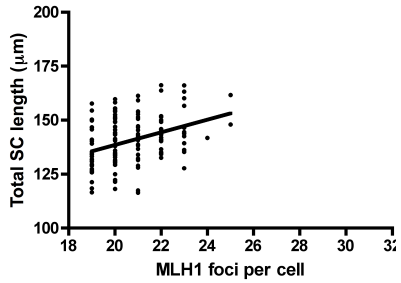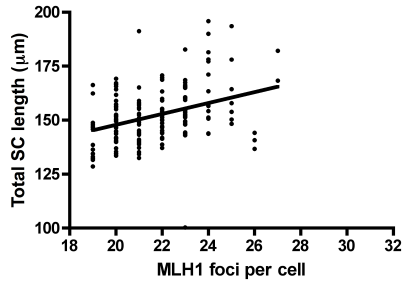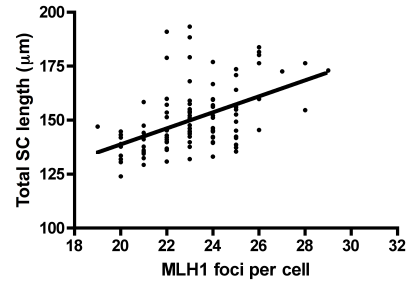

Supplement: S2 Fig — Data points represent total MLH1 foci (x-axis) and corresponding SC length (y-axis) for pachytene cells in 20 dpp, 12 week, and 1 year-old CD-1 males neonatally exposed to EE from 1–12 dpp. Pearson correlation coefficients were calculated to determine relationship between recombination and synaptonemal complex length. For CD-1, the Pearson correlation coefficients were 0.57 (p<0.0001) for placebo and 0.36 (p<0.0001) for 0.25 ng EE-exposed males at 20 dpp and 0.37 (p<0.0001) for placebo and 0.34 (p<0.0001) for 0.25 ng EE-exposed males at 12 weeks old, and 0.35 (p<0.0001) for placebo and 0.48 (p<0.0001) for 0.25 ng EE-exposed males at 1 year old. (PDF) [file pgen.1004949.s005.pdf]
